# Supplementary material for: Metal-dependent and metal-free mechanisms of peptide condensate catalysts
Source: Nat Commun. 2026 Mar 28;17:4548. doi: 10.1038/s41467-026-71117-4 (PMC13194831; doi:10.1038/s41467-026-71117-4)
Supplement: Supplementary file 1 — Supplementary Information [file 41467_2026_71117_MOESM1_ESM.pdf]

Supplementary Information

Metal-dependent and metal-free mechanisms of peptide condensate catalysts

Tlalit Massarano[1,^], Yuqin Yang[2,^], Avigail Baruch Leshem[1], Ori Eran[1], Xiaoyu Wang[2], Hao Dong[2,*] and Ayala Lampel[1,3-5,*]

[^]Equally contributing authors
*co-corresponding authors

[1]Shmunis School of Biomedicine and Cancer Research, George S. Wise Faculty of Life Sciences, Tel Aviv University, Tel Aviv 69978, Israel.
[2]State Key Laboratory of Analytical Chemistry for Life Science, Kuang Yaming Honors School, Chemistry and Biomedicine Innovation Center (ChemBIC), ChemBioMed Interdisciplinary Research Center at Nanjing University, & Institute for Brain Sciences, Nanjing University; Nanjing 210023, China.
[3]Center for Nanoscience and Nanotechnology, Tel Aviv University, Tel Aviv 69978, Israel.
[4]Center for the Physics and Chemistry of Living Systems, Tel Aviv University, Tel Aviv 69978, Israel.
[5]Leibniz Institute of Polymer Research Dresden Max Bergmann Center of Biomaterials Dresden 01069 Dresden, Germany

email: ayalalampel@tauex.tau.ac.il ; donghao@nju.edu.cn

Supplementary Figures

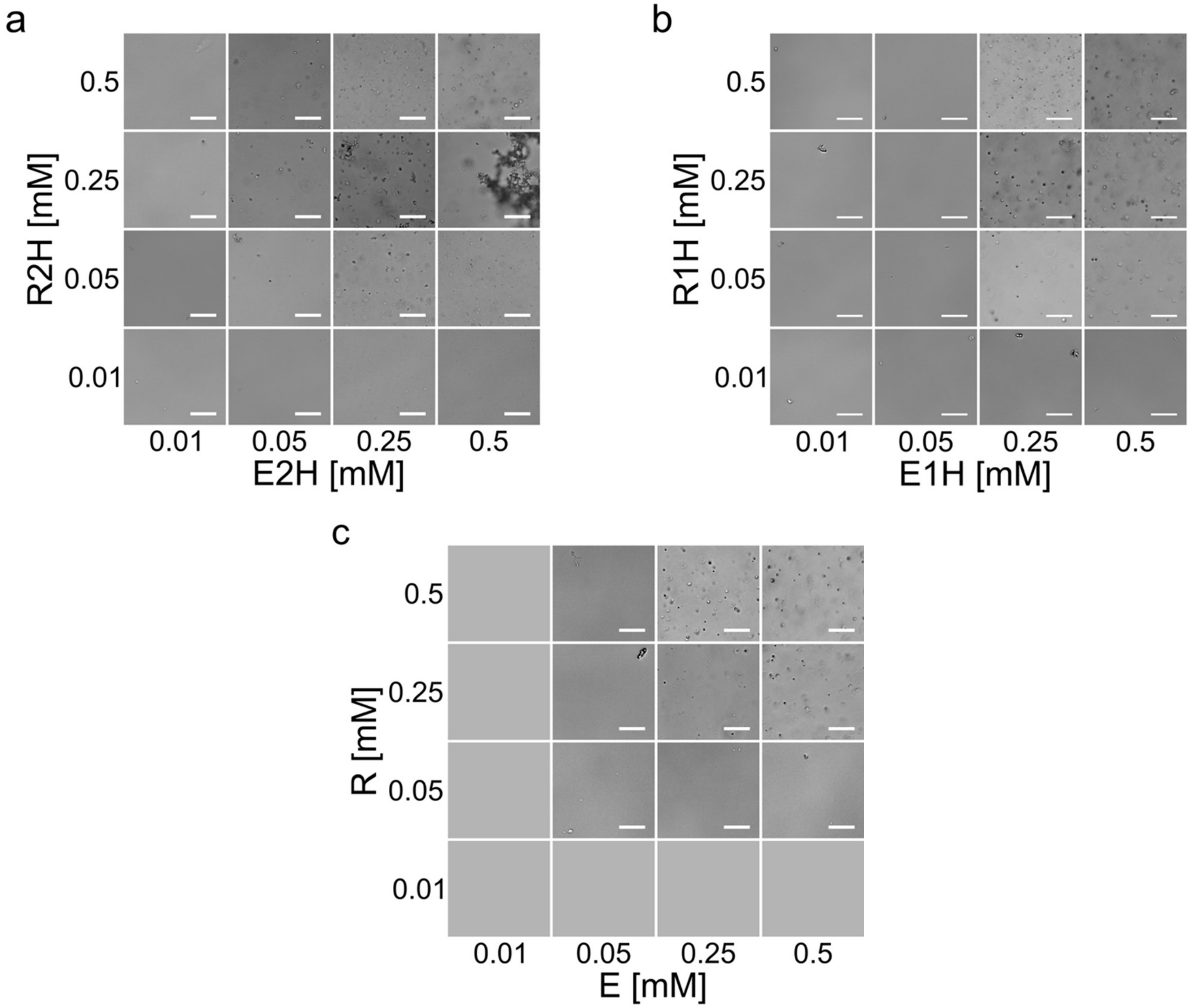

Supplementary Figure 1. Characterization of LLPS propensity using brightfield microscopy as a function of peptide concentration in 10 mM Tris-HCl buffer at pH 7.5 with 0.67 mM $ZnCl_2$. Scale bars=20 μm. The experiment was independently repeated twice.

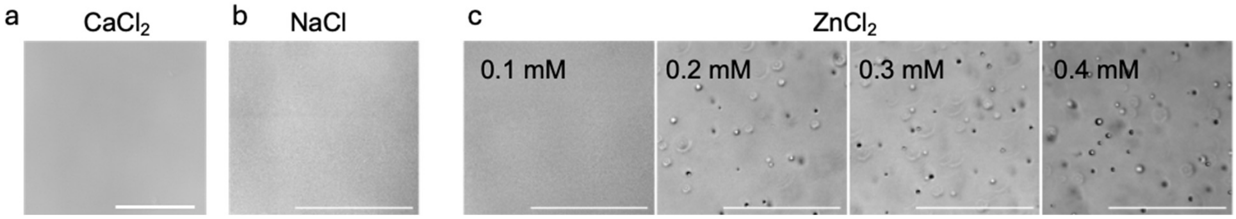

Supplementary Figure 2. Bright field microscopy images of condensates formed by R2H/E2H at 1:1 ratio with final concentration of 0.5 mM, in 10 mM Tris-HCl buffer pH 7.5 with (a) 0.67 mM of $CaCl_2$ or (b) NaCl or (c) increasing concentrations $ZnCl_2$. Scale bars = 50 µm. The experiment was independently repeated twice.

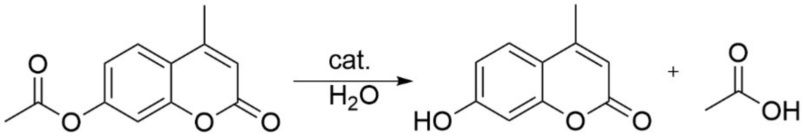

Supplementary Figure 3. Reaction scheme of 4-MU-Ac hydrolysis to 4-MU.

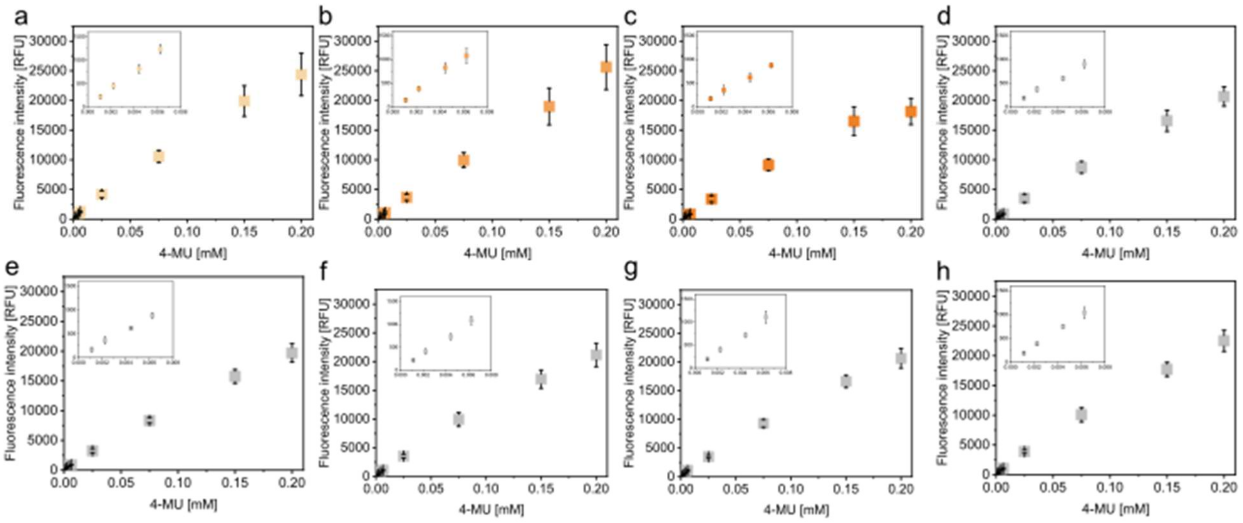

Supplementary Figure 4. Calibration curves of 4-MU in (**a**) R2H/E2H condensates with 0.20 mM $ZnCl_2$, (**b**) R2H/E2H condensates with 0.33 mM $ZnCl_2$, (**c**) R2H/E2H condensates with 0.67 mM $ZnCl_2$, (**d**) R2H with 0.67 mM $ZnCl_2$, (**e**) E2H with 0.67 mM $ZnCl_2$ and (**f**) buffer with 0.20 mM $ZnCl_2$, (**g**) buffer with 0.33 mM $ZnCl_2$ and (**h**) buffer with 0.67 mM $ZnCl_2$. Values represent averages, error bars represent S.D.

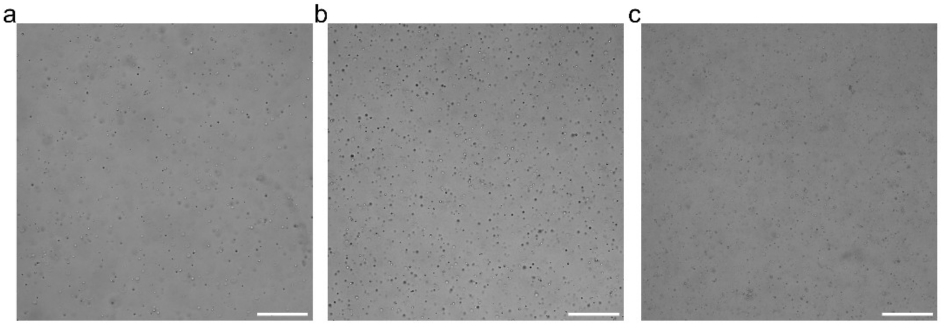

Supplementary Figure 5. Bright field microscopy images of **(a)** R2H/E2H condensates with 0.20 mM $ZnCl_2$, **(b)** R2H/E2H condensates with 0.33 mM $ZnCl_2$ and **(c)** R2H/E2H condensates with 0.67 mM $ZnCl_2$, all in 2.8% MeCN in 10 mM Tris-HCl buffer. Scale bars=50 μm. The experiment was independently repeated twice for 0.20 and 0.33 mM $ZnCl_2$ and three times for 0.67 mM $ZnCl_2$.

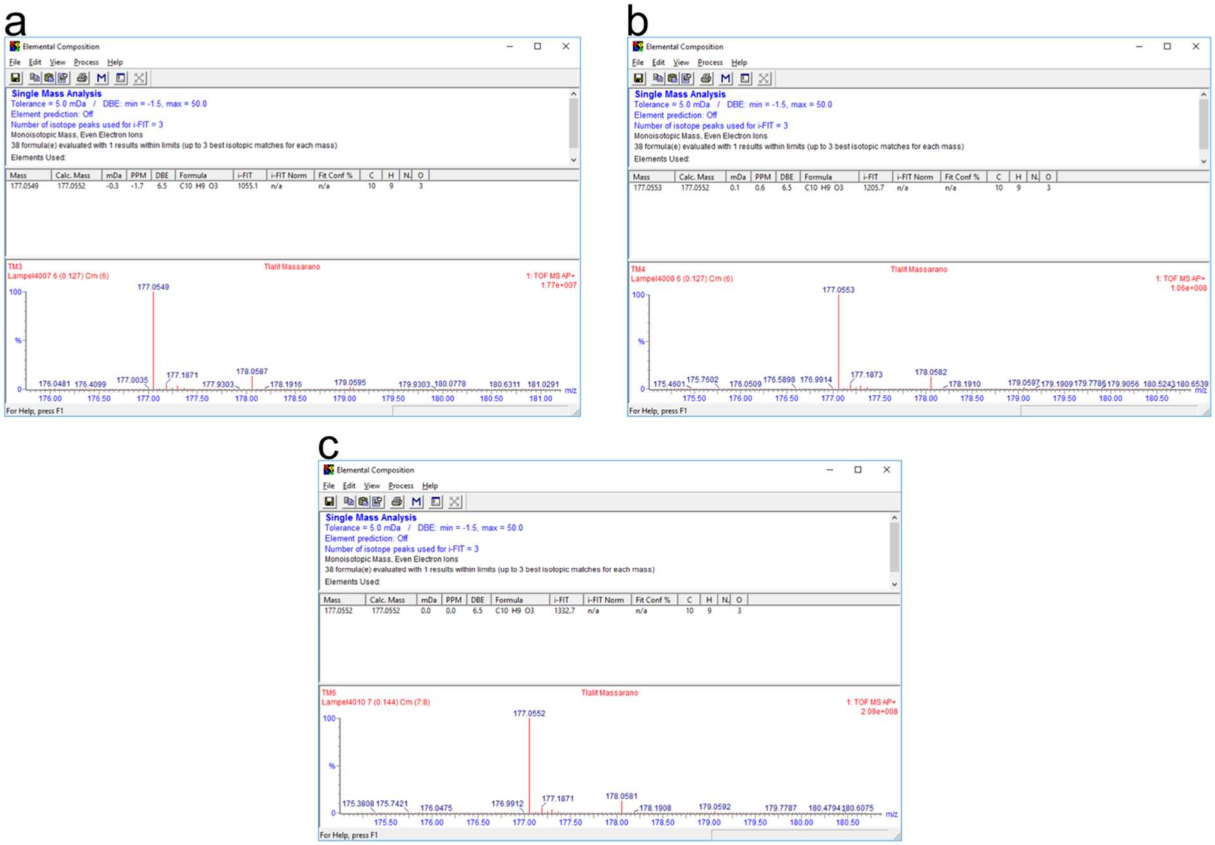

Supplementary Figure 6. APCI– MS analysis of reactions in condensates. APCI–TOF MS showing signals corresponding to the reaction product after addition of 0.7 mM 4-MU-Ac to reaction mixtures **(a)** R2H/E2H + 0.20 mM $ZnCl_2$ condensates, **(b)** R2H/E2H + 0.33 mM $ZnCl_2$ condensates and **(c)** R2H/E2H + 0.67 mM $ZnCl_2$ condensates.

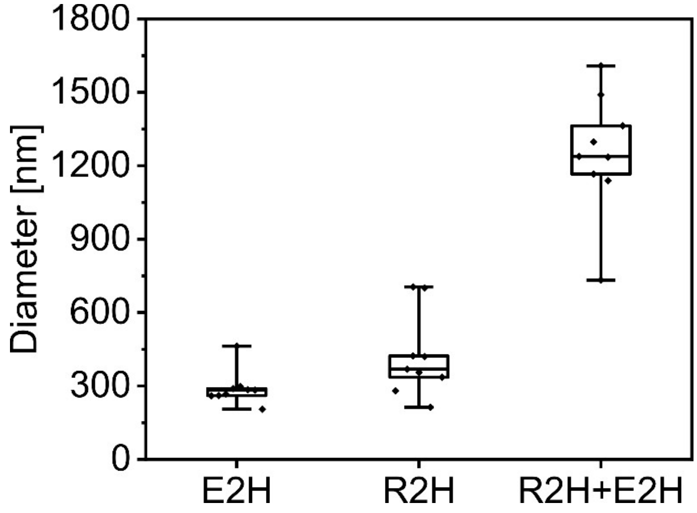

Supplementary Figure 7. DLS analysis of the R2H and E2H peptides and the combined LLPS system R2H/E2H in 10 mM Tris-HCl buffer pH 7.5 with 0.67 mM $ZnCl_2$. Box hinges indicate the first and third quartiles of the corresponding data, mid lines represent medians, and whiskers span the range of the data. Individual data points are shown. Values represent n=9 from 3 independent analyses.

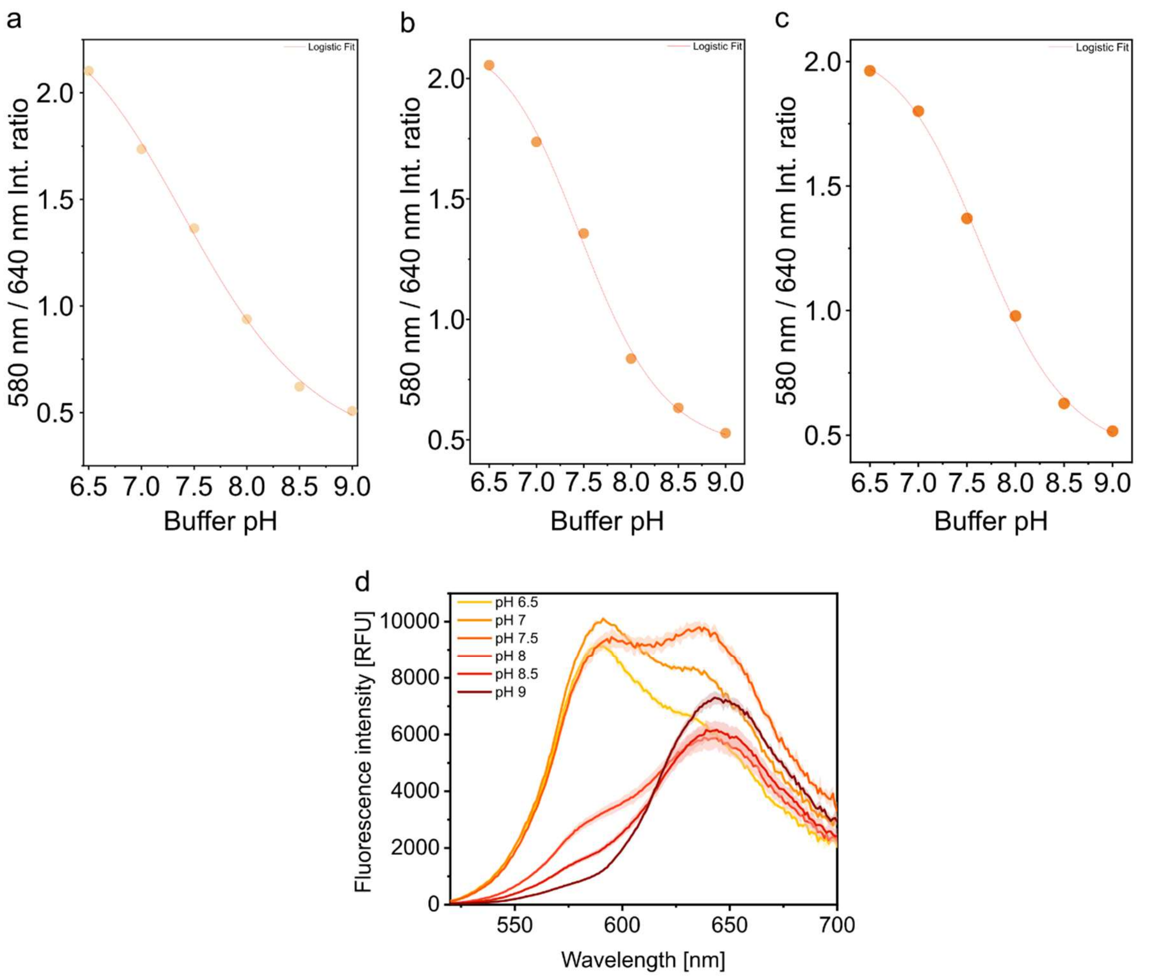

Supplementary Figure 8. a-c. SNARF-1 calibration curves of R2H/E2H condensates with (**a**) 0.20 mM $ZnCl_2$, (**b**) 0.33 mM $ZnCl_2$, and (**c**) 0.67 mM $ZnCl_2$. Red lines indicate the logistic fit. **d.** Emission spectra of the ratiometric pH probe SNARF-1 in Tris buffer with 0.67 mM $ZnCl_2$ at varying pH, showing how the intensity of the two maxima (λ_{em}=580 and 640 nm, λ_{ex}=488 nm) changes as a factor of pH.

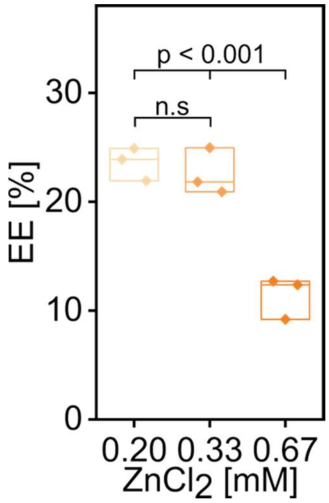

Supplementary Figure 9. 4-MU partitioning in R2H/E2H condensates at varying $ZnCl_2$ concentration. EE% values were obtained by quantifying the dilute-phase concentration of 4-MU following centrifugation and using absorbance spectroscopy based on 4-MU calibration curve. Box hinges indicate the first and third quartiles of the corresponding data, mid lines represent medians, and whiskers span the range of the data. Individual data points are shown. n = 3 from 3 independent measurements.

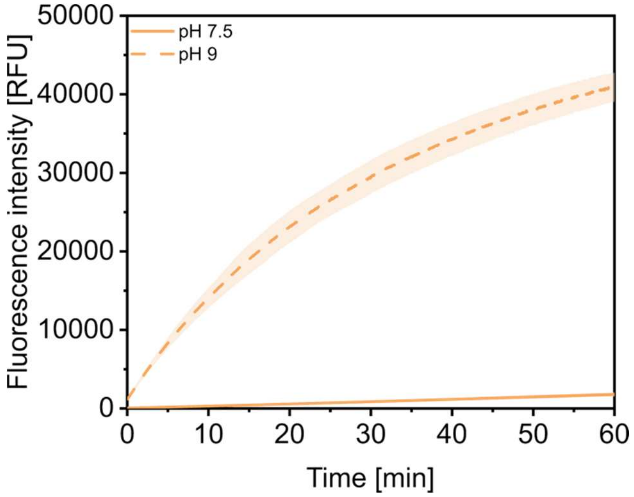

Supplementary Figure 10. Kinetics of 0.7 mM 4-MU-Ac hydrolysis in 0.67 mM $ZnCl_2$-conatining buffer at pH 7.5 and 9.0. Shaded areas represent ± S.D.

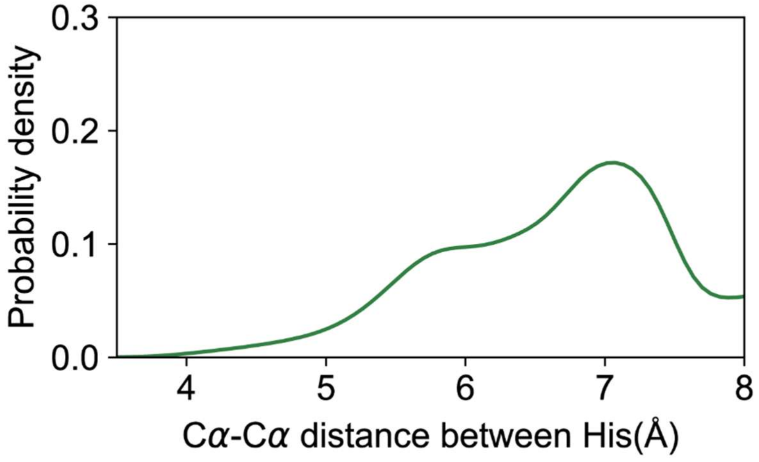

Supplementary Figure 11. The probability density of the distance between the C_α atoms of the two His residues in R2H/E2H system (with the presence of Zn^{2+} ions) observed in MD simulations.

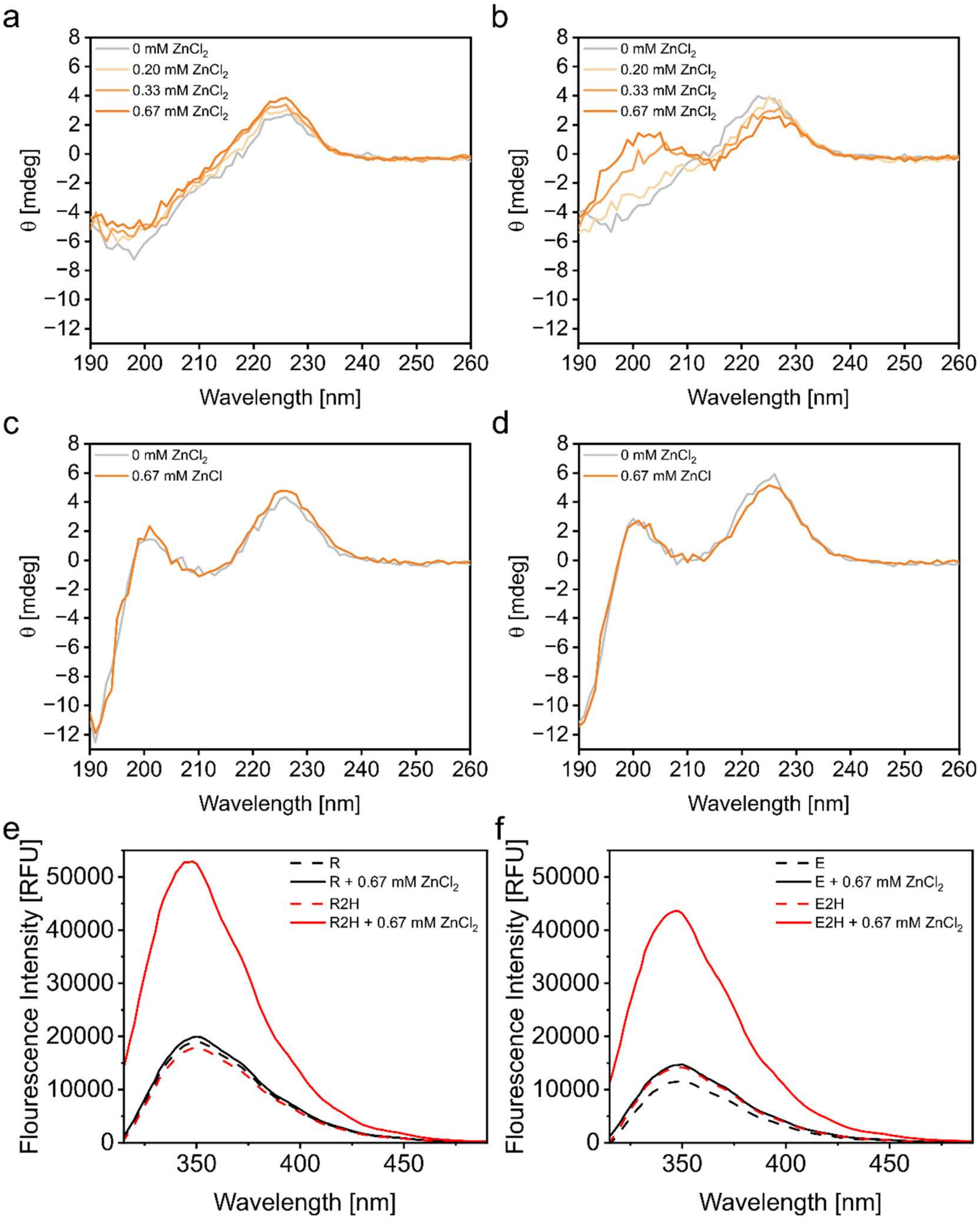

Supplementary Figure 12. **CD and Trp emission analyses showing the interaction of Zn^{2+} ions with His-containing peptides. a-d.** CD analysis of R2H (**a**), E2H (**b**), R (**c**) and E (**d**) with and without $ZnCl_2$. **e-f.** Fluorescence spectroscopy measurements of Trp emission (λ_{ex}=280 nm) of R2H and R (**e**) and E2H and E (**f**). For both analyses, peptide concentration is 0.5 mM in 10 mM Tris-HCl buffer at pH=7.50. Values represent averages of n=3.

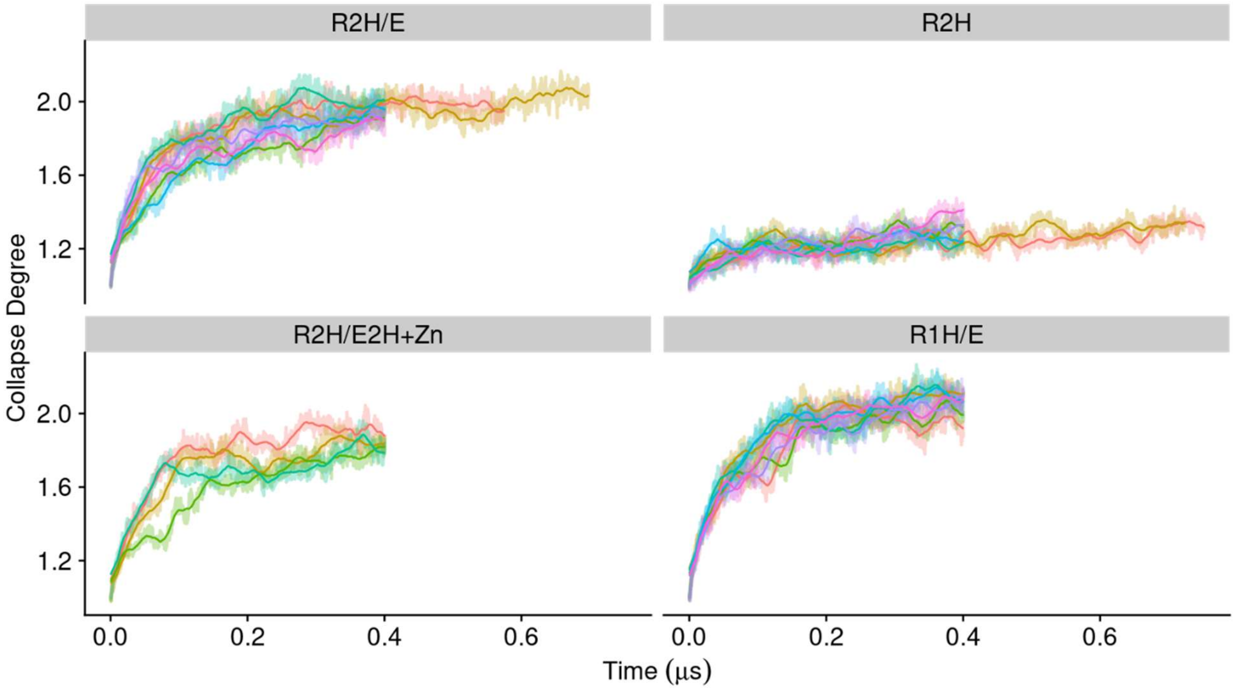

Supplementary Figure 13. Temporal evolution of peptide collapse degree across the simulated systems. Individual trajectories from independent replicas are distinguished by color. Light, semi-transparent traces correspond to the raw data, whereas dark, opaque traces represent the smoothed data.

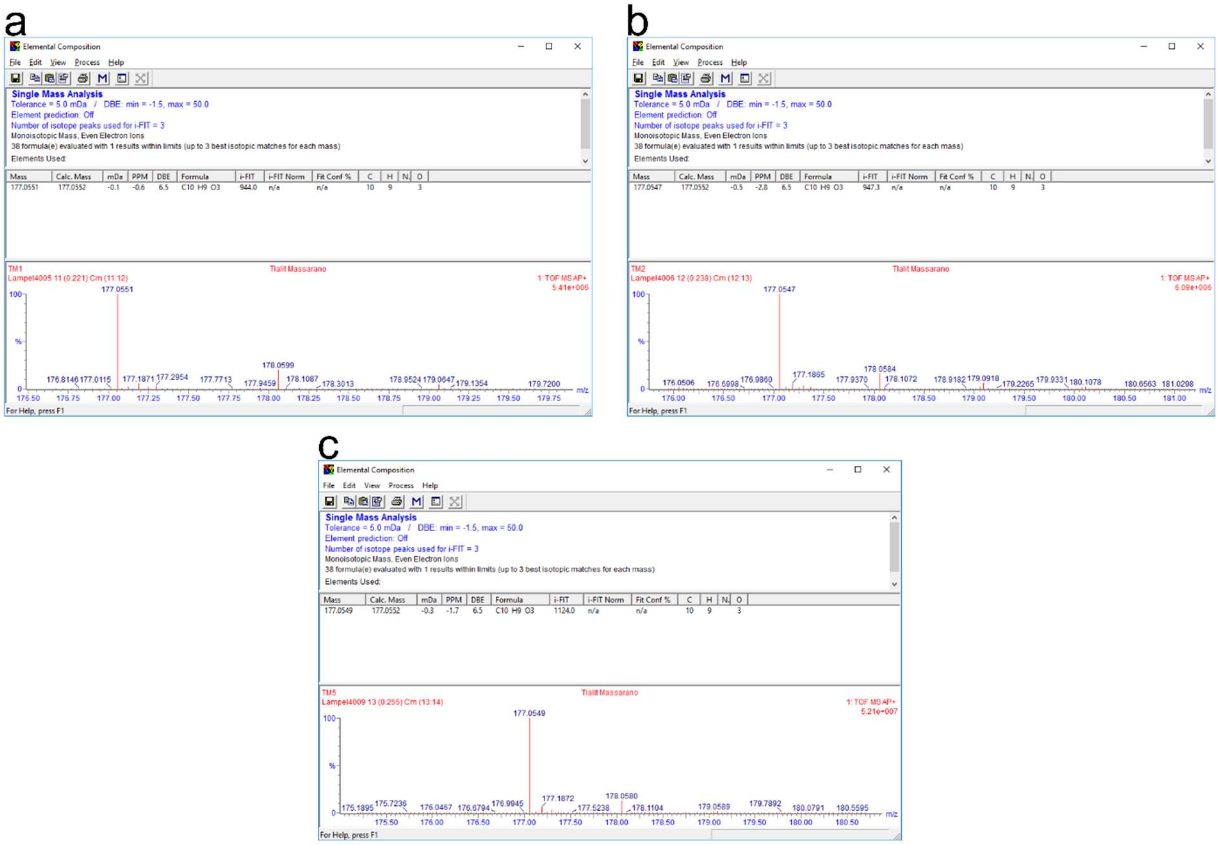

Supplementary Figure 14. APCI– MS analysis of reaction samples. APCI–TOF MS showing signals corresponding to the reaction product after addition of 0.7 mM 4-MU-Ac to reaction mixtures **(a)** R2H/E condensates, **(b)** R1H/E condensates and **(c)** R/E condensates.

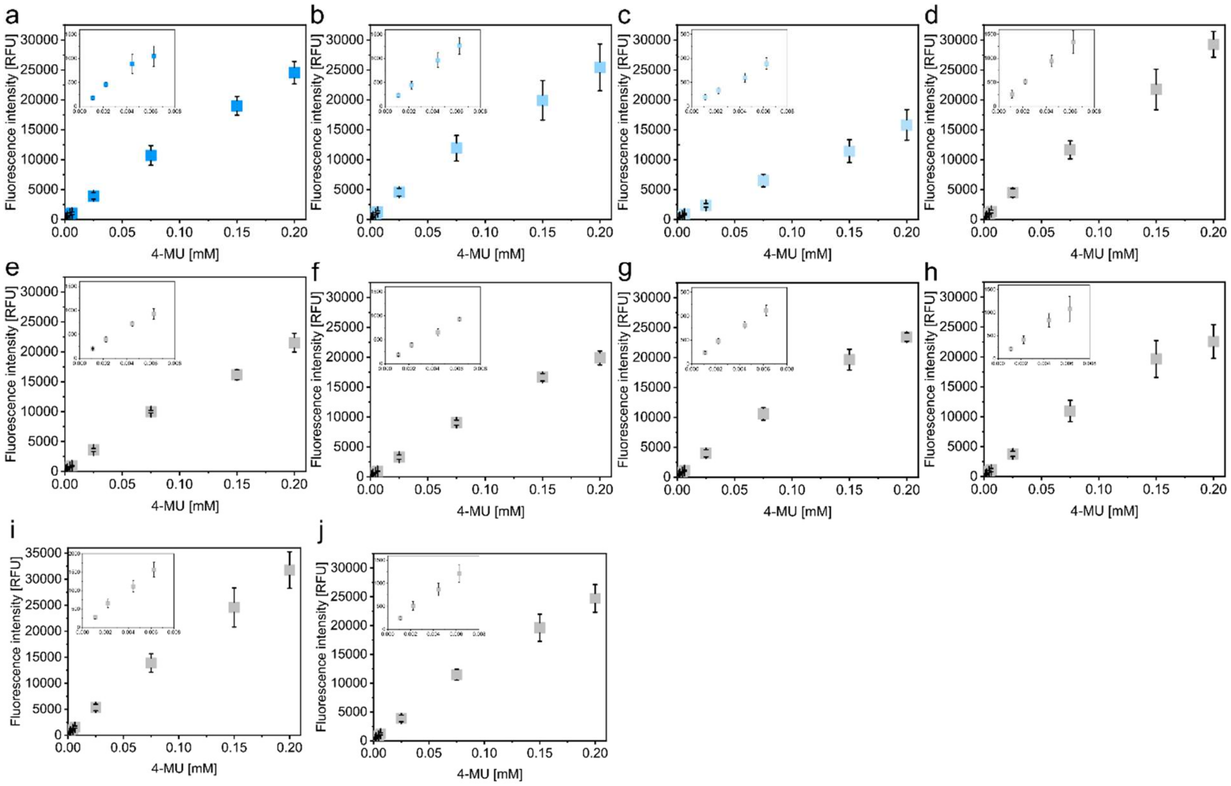

Supplementary Figure 15. Calibration curves of 4-MU in **(a)** R2H/E condensates, **(b)** R1H/E condensates, **(c)** R/E condensates, **(d)** R2H, **(e)** R1H, **(f)** R, **(g)** E, **(h)** His, **(i)** buffer and **(j)** buffer for dilute phase analysis. Values represent averages, error bars represent S.D.

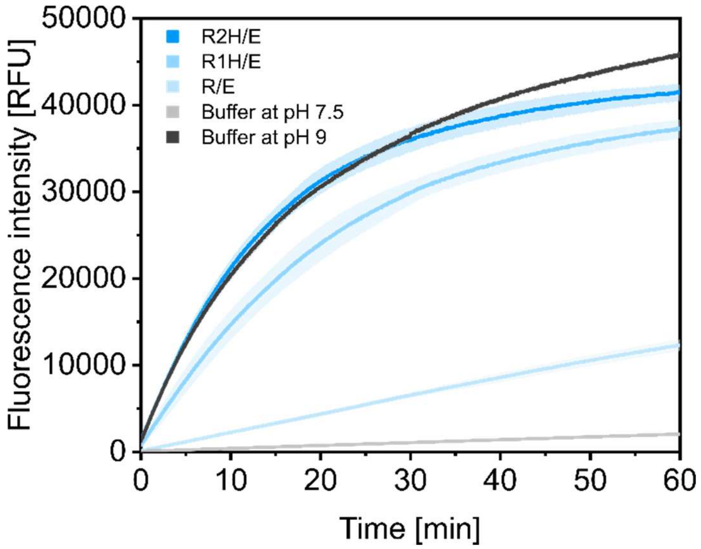

Supplementary Figure 16. Fluorescence intensity increases over time after addition of 0.7 mM 4-MU-Ac to R2H/E, R1H/E and R/E condensates in 10 mM Tris buffer at pH = 7.50 ± 0.04 and to 10 mM Tris buffer at pH = 7.50 ± 0.04 and pH = 9.00 ± 0.04. Values represent averages, shaded areas represent ± S.D.

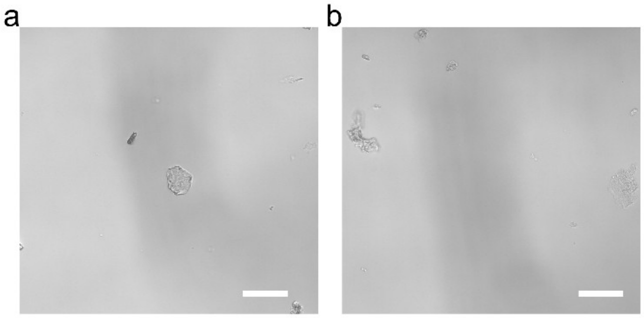

Supplementary Figure 17. Brightfield microscopy images of **(a)** K2H/E mixture and **(b)** K/E mixture. The concentration of each peptide is 10 mM. All samples are in 10 mM Tris buffer at pH=7.50, scale bar=50 μm. The experiment was independently repeated twice.

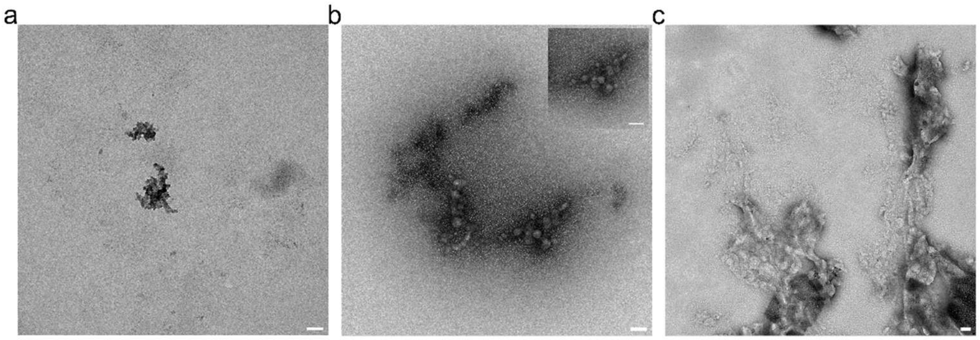

Supplementary Figure 18. Transmitted electron microscopy micrographs of **(a)** 2.8% MeCN in 10 mM Tris buffer at pH = 7.50 ± 0.04 and **(b-c)** 3 mM R2H with 2.8% MeCN in 10 mM Tris buffer at pH=7.5. Scale bar = 100 nm. The experiment was independently repeated twice for R2H and once for buffer.

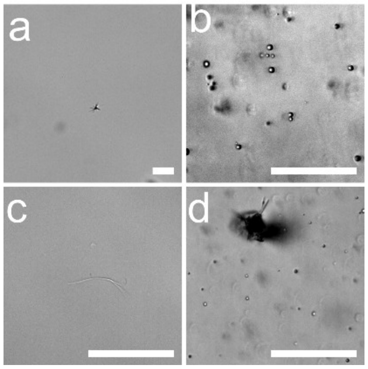

Supplementary Figure 19. Bright field microscopy images of (**a**) 0.7 mM 4-MU-Ac in R2H soluble solution at 3 min, (**b**) 0.7 mM 4-MU-Ac in R2H/E condensates solution at 15 min, (**c**) 1.1 mM 4-MU-Ac in R2H soluble solution immediately after addition and (**d**) 1.1 mM 4-MU-Ac in R2H/E condensates solution at 5 min. Scale bars=50 µm. Each experiment repeated itself three times.

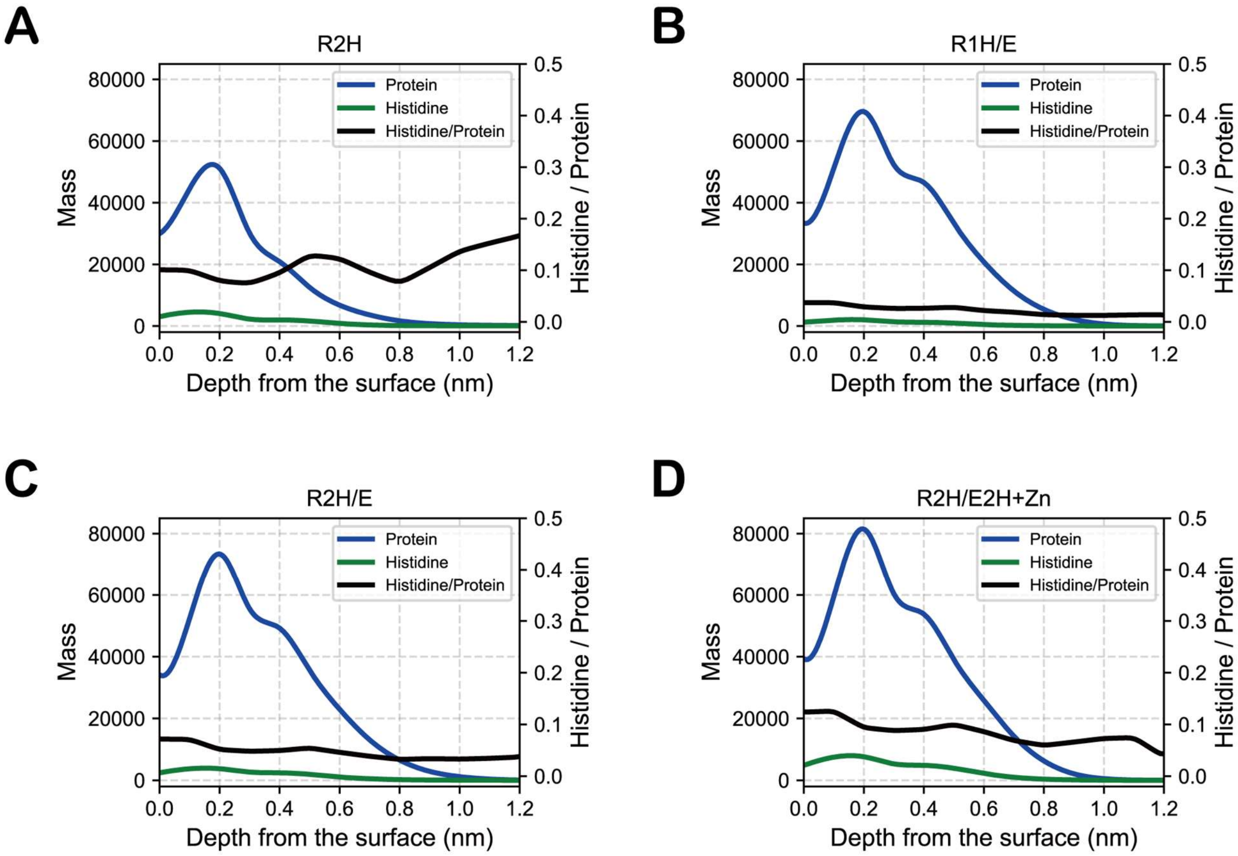

Supplementary Figure 20. **Radial distribution of peptides and histidine within the droplet**. Plotted as a function of distance from the droplet interface (0 nm corresponds to the interface), the blue curve shows the atomic number density profile of peptides, and the green curve shows that of histidine. The black curve represents the histidine-to-peptide ratio, highlighting its near-constancy within the 0–1.2 nm range, consistent with the homogeneous distribution discussed in the text.

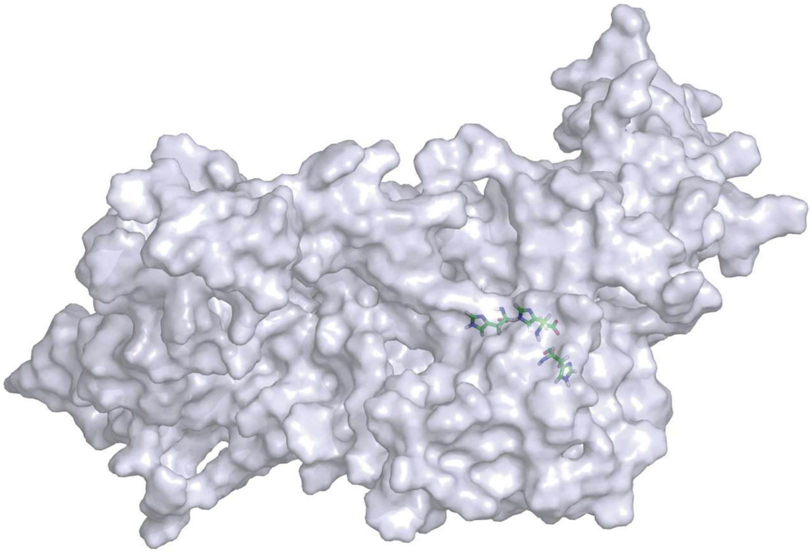

Supplementary Figure 21. Snapshot of histidine distribution within the peptide cluster. The peptide cluster is shown as a surface representation, and histidine residues are depicted in stick format.

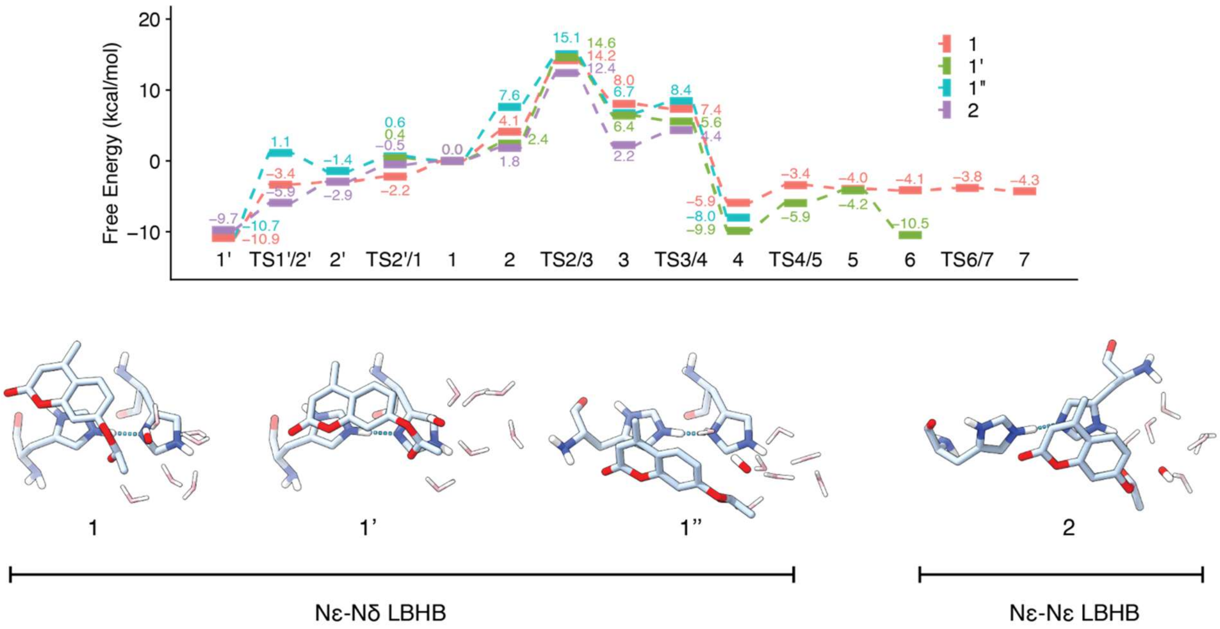

Supplementary Figure 22. The free energy profiles for 4-Mu-Ac hydrolysis under metal-free conditions. The free energy profiles under various substrate binding orientations and two different hydrogen-bonding modes between the His are presented, along with the **TS2/3** transition state structures for each pathway.

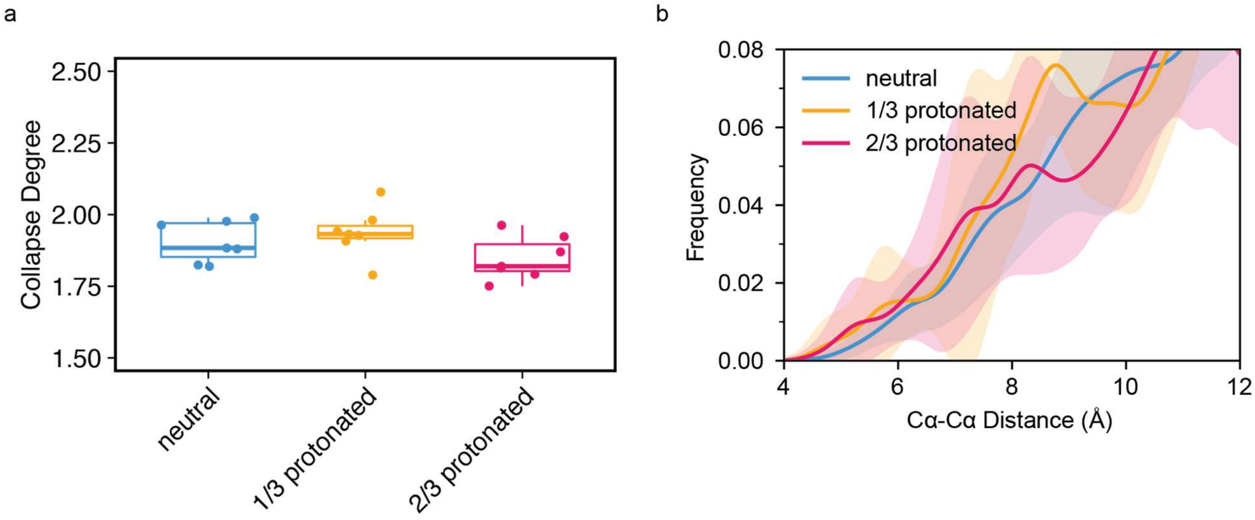

Supplementary Figure 23. Simulation results of the R2H/E system upon histidine protonation. Comparisons were made between the fully neutral system and systems where 1/3 or 2/3 of the R2H chains contain a single protonated histidine. The panels display (a) the collapse degree (n = 7 independent trajectories) and (b) the pairwise histidines' Cα-Cα distance distribution. Box hinges indicate the first and third quartiles of the corresponding data, mid lines represent medians, and whiskers span the range of the data.

Supplementary Tables

Notation	Sequence
K2H	WGKGKGKGWPGVGHGH
K	WGKGKGKWPGVGY

Supplementary Table 1. Designed LLPS-promoting peptide sequences and their notations.

R2H	0.5	0.00825	0.25825	1.03875	1.536
	0.25	0	0.23275	0.9645	1.3475
	0.05	0.00825	0.1155	0.79325	0.8705
	0.01	0.00625	0.03175	0.1915	0.2405
		0.01	0.05	0.25	0.5
		E2H			

Supplementary Table 2. Quantitative data used to generate the phase diagram heat maps shown in Fig. 2a.

R1H	0.5	0	0	1.42125	1.43175
	0.25	0	0	1.0555	1.25025
	0.05	0	0	0.24625	0.40375
	0.01	0	0	0	0
		0.01	0.05	0.25	0.5
		E1H			

Supplementary Table 3. Quantitative data used to generate the phase diagram heat maps shown in Fig. 2b.

R	0.5	0.00025	0.825	1.1845
	0.25	0	1.02075	0.61625
	0.05	0	0	0
		0.05	0.25	0.5
		E		

Supplementary Table 4. Quantitative data used to generate the phase diagram heat maps shown in Fig. 2c.

System	V_0 [mM/sec]	S.D.
**buffer**	3.40313E-06	1.37707E-06
**R2H/E2H with 0.20 mM Zn**	6.5837E-05	7.0019E-06
**R2H/E2H with 0.33 mM Zn**	5.13679E-05	5.45282E-06
**R2H/E2H with 0.67 mM Zn**	1.96469E-05	1.88366E-06
**R2H with 0.67 mM Zn**	1.03904E-05	6.16059E-07
**E2H with 0.67 mM Zn**	5.32016E-06	6.39036E-07
**buffer with 0.20 mM Zn**	5.29573E-06	1.71083E-06
**buffer with 0.33 mM Zn**	3.18474E-06	7.58202E-07
**buffer with 0.67 mM Zn**	3.01156E-06	5.48586E-07

Supplementary Table 5. Catalytic activity (V_0) of Zn^{2+}-containing LLPS systems with 0.7 mM 4-MU-Ac including control samples of soluble peptides (0.5 mM) in bulk buffer with Zn^{2+}, and peptide-free Zn^{2+}-containing buffers. Values represent averages and S.D. The sample size and number of independent repeats are described in Supplementary Table 11.

	pH (Condensates)	n (valid/total)	pH (Dilute phase)	n (valid/total)
**R2H/E2H with 0.20 mM Zn***	10.8±0.6	6/10	7.7±0.1	10/10
**R2H/E2H with 033 mM Zn***	N.D	2/12	7.7±0.1	11/11
**R2H/E2H with 0.67 mM Zn**	8.5±0.1	14/14	7.6±0.4	13/13
**R2H/E***	N.D	0/12	8.0±0.1	12/12
**R/E**	9.5±0.3	30/30	8.4±0.3	27/27

Supplementary Table 6. Ratiometric pH measurements in condensates and dilute phase. Ratiometric pH values measured using SNARF-1 in condensates and dilute phase under different Zn^{2+} concentrations and peptide compositions. Values represent averages and S.D. *N.D. (Not Determined): Fluorescence intensity ratios fell below the lower asymptote (R_{min}) of the logistic calibration curve, consistent with pH values exceeding the probe's reliable dynamic range.

System	V_{max} [M·sec^{-1}]	k_{cat}[sec^{-1}]	K_m[M]	ε[sec^{-1}·M^{-1}]
**R/E**	$2.13\cdot10^{-07} \pm 3.98\cdot10^{-08}$	$7.09\cdot10^{-05} \pm 1.31\cdot10^{-05}$	$3.24\cdot10^{-03} \pm 6.07\cdot10^{-04}$	0.02 ± 0.01
**R1H/E**	$1.49\cdot10^{-06} \pm 3.98\cdot10^{-07}$	$4.97\cdot10^{-04} \pm 1.33\cdot10^{-04}$	$3.95\cdot10^{-03} \pm 1.06\cdot10^{-03}$	0.13 ± 0.05
**R2H/E**	$7.45\cdot10^{-07} \pm 9.20\cdot10^{-08}$	$2.48\cdot10^{-04} \pm 3.07\cdot10^{-05}$	$1.15\cdot10^{-03} \pm 1.55\cdot10^{-04}$	0.22 ± 0.04
**R2H (soluble)**	$7.58\cdot10^{-07} \pm 1.67\cdot10^{-07}$	$2.53\cdot10^{-04} \pm 5.57\cdot10^{-05}$	$1.43\cdot10^{-03} \pm 3.35\cdot10^{-04}$	0.18 ± 0.06

Supplementary Table 7. Kinetic parameters calculated for R2H/E, R1H/E and R/E with the corresponding calculated errors. Values represent average and corresponding calculated errors are presented. The sample size and number of independent repeats are described in Supplementary Table 11.

System	V_0 [mM/sec]	S.D.
**R2H/E**	0.000275912	3.45078E-05
**R2H/E dilute phase**	0.000152650	1.97367E-05
**R1H/E**	0.000215284	2.25863E-05
**R1H/E dilute phase**	0.000127961	1.28622E-05
**R/E**	4.02577E-05	5.04805E-06
**R/E dilute phase**	6.72419E-06	9.81696E-07
**R2H**	0.000251438	2.95355E-05
**R1H**	0.000219724	1.35146E-05
**R**	1.48912E-05	1.30279E-06
**E**	6.13226E-06	7.00297E-07
**His**	4.97058E-05	6.1845E-06
**buffer**	3.40313E-06	1.37707E-06

Supplementary Table 8. Catalytic activity (V_0) of Zn^{2+}-free LLPS systems with 0.7 mM 4-MU-Ac, including control samples of soluble peptides (R2H, E2H, R, E), dilute phase and free His in bulk buffer, and peptide-free buffer. The concentration of the soluble peptides and His is 3 mM. Values represent averages, S.D. are presented. The sample size and number of independent repeats are described in Supplementary Table 11.

System	No. neutral R2H	No. R2H with one protonated His	No. E
**R2H/E neutral**	20	0	20
**R2H/E 1/3 protonated**	13	7	20
**R2H/E 2/3 protonated**	6	14	20

Supplementary Table 9. Summary of simulation systems with different protonation states.

System	Box	No. peptide	No. Cl^-	No. Zn^{2+}	No. water	No. atoms
R2H	12.7 × 12.7 × 12.7 nm^3	20 R2H	60	0	67313	206719
R2H/E	12.7 × 12.7 × 12.7 nm^3	20 R2H & 20 E	0	0	66105	206395
R2H/E2H+Zn	12.7 × 12.7 × 12.7 nm^3	20 R2H & 20 E2H	370	185	65316	205403
R1H/E	12.7 × 12.7 × 12.7 nm^3	20 R1H & 20 E	0	0	66244	206332

Supplementary Table 10. Details of the simulated systems.

**Fig. no.**	**Experiment description**	**Statistical Methods**	**Sample numbers/Experimental replicates**
2a-c	Turbidity measurements		Representative results of n = 4 wells. This was repeated twice.
2d	% total peptide in the dense phase	One-way ANOVA (two-sided) (OriginPro 2025) was used to assess differences between groups. Homogeneity of variances was evaluated using Levene's test (α = 0.05) and was not violated. Statistical significance was assessed at α = 0.05 (F(2,6) = 106.20). 0.33-0.20 [0.09, 16.92]; 0.67-0.20 [29.66, 46.49]; 0.67-0.33 [21.15, 37.99]. Post hoc comparisons were performed using Tukey's test, as indicated in the figure.	For all systems: n = 3 from 3 independent measurements.
2e	Reaction kinetics	±25% cutoff	**R2H/E2H + 0.20 mM $ZnCl_2$:** 0.3 n = 14 wells from 4 independent measurements; 0.5 n = 12 wells from 3 independent measurements; 0.7 n = 12 wells from 3 independent measurements; 0.9 n = 11 wells from 3 independent measurements; 1.1 n = 16 wells

Fig. no.	Experiment description	Statistical Methods	Sample numbers/Experimental replicates
			from 4 independent measurements.<br>**R2H/E2H + 0.33 mM $ZnCl_2$**: 0.3 n = 15 wells from 4 independent measurements; 0.5 n = 11 wells from 3 independent measurements; 0.7 n = 12 wells from 3 independent measurements; 0.9 n = 11 wells from 3 independent measurements; 1.1 n = 11 wells from 3 independent measurements.<br>**R2H/E2H + 0.67 mM $ZnCl_2$:** 0.3 n = 11 wells from 3 independent measurements; 0.5 n = 11 wells from 3 independent measurements; 0.7 n = 12 wells from 3 independent measurements; 0.9 n = 12 wells from 3 independent measurements; 1.1 n = 12 wells from 3 independent measurements.
Supplementary Figure 4a	Calibration curve	±30% cutoff	R2H/E2H + 0.20 mM $ZnCl_2$: 0.2 n = 11 wells from 3 independent measurements; 0.15 n = 12 wells from 3 independent measurements; 0.075 n = 11 wells from 3 independent measurements; 0.025 n = 11 wells from 3 independent measurements; 0.006 n = 12 wells from 3 independent measurements; 0.004 n = 12 wells from 3 independent measurements; 0.002 n = 12 wells from 3 independent

Fig. no.	Experiment description	Statistical Methods	Sample numbers/Experimental replicates
			measurements; 0.001 n = 11 wells from 3 independent measurements.
Supplementary Figure 4b	Calibration curve	±30% cutoff	R2H/E2H + 0.33 mM $ZnCl_2$: 0.2 n = 12 wells from 3 independent measurements; 0.15 n = 11 wells from 3 independent measurements; 0.075 n = 12 wells from 3 independent measurements; 0.025 n = 12 wells from 3 independent measurements; 0.006 n = 8 wells from 2 independent measurements; 0.004 n = 8 wells from 2 independent measurements; 0.002 n = 8 wells from 2 independent measurements; 0.001 n = 7 wells from 2 independent measurements.
Supplementary Figure 4c	Calibration curve	±30% cutoff	R2H/E2H + 0.67 mM $ZnCl_2$: 0.2 n = 7 wells from 2 independent measurements; 0.15 n = 8 wells from 2 independent measurements; 0.075 n = 7 wells from 2 independent measurements; 0.025 n = 5 wells from 2 independent measurements; 0.006 n = 7 wells from 2 independent measurements; 0.004 n = 8 wells from 2 independent measurements; 0.002 n = 7 wells from 2 independent measurements; 0.001 n = 7 wells from 2 independent measurements.

Fig. no.	Experiment description	Statistical Methods	Sample numbers/Experimental replicates
Supplementary Figure 4d	Calibration curve	±30% cutoff	R2H + 0.67 mM $ZnCl_2$: 0.2 n = 8 wells from 2 independent measurements; 0.15 n = 8 wells from 2 independent measurements; 0.075 n = 8 wells from 2 independent measurements; 0.025 n = 8 wells from 2 independent measurements; 0.006 n = 8 wells from 2 independent measurements; 0.004 n = 7 wells from 2 independent measurements; 0.002 n = 7 wells from 2 independent measurements; 0.001 n = 6 wells from 2 independent measurements.
Supplementary Figure 4e	Calibration curve	±30% cutoff	E2H + 0.67 mM $ZnCl_2$: 0.2 n = 8 wells from 2 independent measurements; 0.15 n = 8 wells from 2 independent measurements; 0.075 n = 6 wells from 2 independent measurements; 0.025 n = 7 wells from 2 independent measurements; 0.006 n = 8 wells from 2 independent measurements; 0.004 n = 8 wells from 2 independent measurements; 0.002 n = 8 wells from 2 independent measurements; 0.001 n = 8 wells from 2 independent measurements.
Supplementary Figure 4f	Calibration curve	±30% cutoff	Buffer + 0.20 mM $ZnCl_2$: 0.2 n = 8 wells from 2 independent measurements; 0.15 n = 8 wells from 2 independent

Fig. no.	Experiment description	Statistical Methods	Sample numbers/Experimental replicates
			measurements; 0.075 n = 8 wells from 2 independent measurements; 0.025 n = 8 wells from 2 independent measurements; 0.006 n = 8 wells from 2 independent measurements; 0.004 n = 8 wells from 2 independent measurements; 0.002 n = 8 wells from 2 independent measurements; 0.001 n = 8 wells from 2 independent measurements.
Supplementary Figure 4g	Calibration curve	±30% cutoff	Buffer + 0.33 mM $ZnCl_2$: 0.2 n = 7 wells from 2 independent measurements; 0.15 n = 8 wells from 2 independent measurements; 0.075 n = 8 wells from 2 independent measurements; 0.025 n = 8 wells from 2 independent measurements; 0.006 n = 8 wells from 2 independent measurements; 0.004 n = 8 wells from 2 independent measurements; 0.002 n = 8 wells from 2 independent measurements; 0.001 n = 8 wells from 2 independent measurements.
Supplementary Figure 4h	Calibration curve	±30% cutoff	Buffer + 0.67 mM $ZnCl_2$: 0.2 n = 8 wells from 2 independent measurements; 0.15 n = 8 wells from 2 independent measurements; 0.075 n = 8 wells from 2 independent measurements; 0.025 n = 8 wells from 2 independent

Fig. no.	Experiment description	Statistical Methods	Sample numbers/Experimental replicates
			measurements; 0.006 n = 8 wells from 2 independent measurements; 0.004 n = 8 wells from 2 independent measurements; 0.002 n = 8 wells from 2 independent measurements; 0.001 n = 8 wells from 2 independent measurements.
Supplementary Figure 7	DLS		For all systems: n = 9 from 3 independent measurements.
Supplementary Figure 8a-c	SNARF-1 calibration curves		For all systems: n = 5 positions in the well for each pH value.
Supplementary Figure 8d	SNARF-1 spectroscopic measurements	Outliers were excluded based on visual deviation from the main cluster of fluorescence curves	pH 6.5 n = 3 wells; pH 7 n = 3 wells; pH 7.5 n = 4 wells; pH 8 n = 4 wells; pH 8.5 n = 4 wells; pH 9 = 4
2g	SNARF-1 ratiometric pH probe	Condensates: One-way ANOVA (two-sided) (OriginPro 2025) was used to assess differences between groups. Homogeneity of variances was evaluated using Levene's test (α = 0.05) and was not violated. Statistical significance was assessed at α = 0.05 ($F(2,33)$ = 351.36).	Detailed in Table S6

Fig. no.	Experiment description	Statistical Methods	Sample numbers/Experimental replicates
		$0.33_{cond.}$-$0.20_{cond.}$ [0.05, 0.12]; $0.67_{cond.}$-$0.20_{cond.}$ [0.31, 0.38]; $0.67_{cond.}$-$0.33_{cond.}$ [0.23, 0.30].<br>Post hoc comparisons were performed using Tukey's test, as indicated in the figure.<br>Dilute phase: One-way Welch ANOVA (two-sided) (OriginPro 2025) was used to assess differences between groups. Statistical significance was assessed at $\alpha = 0.05$ ($F(2,20) = 2.26$). 0.33_{dilute}-0.20_{dilute} [-0.29, 0.18]; 0.67_{dilute}-0.20_{dilute} [-0.09, 0.36]; 0.67_{dilute}-0.33_{dilute} [-0.03, 0.41].<br>Post hoc comparisons were performed using Games-Howell pairwise comparison, no significant differences were found.	

Fig. no.	Experiment description	Statistical Methods	Sample numbers/Experimental replicates
2i-j	FRAP, $t_{1/2}$, % recovery	2i- A Two-sample Welch's t-test (two-sided) (OriginPro 2025) was used to compare the two groups.<br>Statistical significance was assessed at α = 0.05.<br>t(17.32) = −5.14 with a 95% confidence interval of [-17.77, -8.17].<br>p value is indicated in the figure.	**R2H/E2H+0.20 mM $ZnCl_2$:** 17 condensates from 3 independent measurements.<br>**R2H/E2H+0.33 mM $ZnCl_2$:** 12 condensates from 2 independent measurements.<br>**R2H/E2H+0.67 mM $ZnCl_2$:** 15 condensates from 3 independent measurements.
Supplementary Figure 9	4-MU partitioning in Zn^{2+}-dependent systems	One-way ANOVA (two-sided) (OriginPro 2025) was used to assess differences between groups.<br>Homogeneity of variances was evaluated using Levene's test (α = 0.05) and was not violated.<br>Statistical significance was assessed at α = 0.05 (F(2,6) = 38.91). 0.33-0.20 [-5.70, 3.68]; 0.67-0.20 [-16.84, -7.46]; 0.67-0.33 [-15.83, -6.45]. Post hoc comparisons were	For all systems: n = 3 from 3 independent measurements.

Fig. no.	Experiment description	Statistical Methods	Sample numbers/Experimental replicates
		performed using Tukey's test, as indicated in the figure.	
Supplementary Figure 10	Reaction kinetics analysis for 60 min		n = 4 wells
Supplementary Figure 12a-d	CD		
Supplementary Figure 12e	Trp emission	Outliers were excluded based on visual deviation from the main cluster of fluorescence curves	**R2H+0.67 mM $ZnCl_2$:** n = 10 wells from 3 independent measurements.<br>**R2H:** n = 10 wells from 3 independent measurements.<br>**R+0.67 mM $ZnCl_2$:** n = 10 wells from 3 independent measurements.<br>**R:** n = 11 wells from 3 independent measurements.
Supplementary Figure 12f	Trp emission		**E2H+0.67 mM $ZnCl_2$:** n = 3 wells.<br>**E2H:** n = 3 wells.<br>**E+0.67 mM $ZnCl_2$:** n = 3 wells.<br>**E:** n = 3 wells.
4b	% total peptide in the dense phase	A Two-sample Welch's t-test (two sided) (OriginPro 2025) was used to compare the two groups.<br>Statistical significance was assessed at $\alpha = 0.05$.<br>$t(4.27) = -3.59$, with a 95% confidence interval of [-30.14, -1.62].	R2H/E: n = 3 from 3 independent measurements.; R/E: n = 5 from 5 independent measurements.

Fig. no.	Experiment description	Statistical Methods	Sample numbers/Experimental replicates
		p value is indicated in the figure.	
Supplementary Figure 15a	Calibration curve	±30% cutoff	R2H/E: 0.2 n = 7 wells from 2 independent measurements; 0.15 n = 7 wells from 2 independent measurements; 0.075 n = 8 wells from 2 independent measurements; 0.025 n = 8 wells from 2 independent measurements; 0.006 n = 8 wells from 2 independent measurements; 0.004 n = 7 wells from 2 independent measurements; 0.002 n = 7 wells from 2 independent measurements; 0.001 n = 7 wells from 2 independent measurements.
Supplementary Figure 15b	Calibration curve	±30% cutoff	R1H/E: 0.2 n =12 wells from 3 independent measurements; 0.15 n = 12 wells from 3 independent measurements; 0.075 n = 12 wells from 3 independent measurements; 0.025 n = 11 wells from 3 independent measurements; 0.006 n = 12 wells from 3 independent measurements; 0.004 n = 11 wells from 3 independent measurements; 0.002 n = 11 wells from 3 independent measurements; 0.001 n = 9 wells from 3 independent measurements.
Supplementary Figure 15c	Calibration curve	±30% cutoff	R/E: 0.2 n = 11 from 3 independent measurements; 0.15 n = 12 wells from 3 independent measurements; 0.075 n = 10 wells

Fig. no.	Experiment description	Statistical Methods	Sample numbers/Experimental replicates
			from 3 independent measurements; 0.025 n = 11 wells from 3 independent measurements; 0.006 n = 9 wells from 3 independent measurements; 0.004 n = 12 wells from 3 independent measurements; 0.002 n = 11 wells from 3 independent measurements; 0.001 n = 8 wells from 3 independent measurements.
Supplementary Figure 15d	Calibration curve	±30% cutoff	R2H: 0.2 n = 8 wells from 2 independent measurements; 0.15 n = 8 wells from 2 independent measurements; 0.075 n = 8 wells from 2 independent measurements; 0.025 n = 7 wells from 2 independent measurements; 0.006 n = 8 wells from 2 independent measurements; 0.004 n = 8 wells from 2 independent measurements; 0.002 n = 8 wells from 2 independent measurements; 0.001 n = 5 wells from 2 independent measurements.
Supplementary Figure 15e	Calibration curve	±30% cutoff	R1H: 0.2 n = 8 wells from 2 independent measurements; 0.15 n = 8 wells from 2 independent measurements; 0.075 n = 8 wells from 2 independent measurements; 0.025 n = 8 wells from 2 independent measurements; 0.006 n = 8 wells from 2 independent measurements; 0.004 n = 7 wells

**Fig. no.**	**Experiment description**	**Statistical Methods**	**Sample numbers/Experimental replicates**
			from 2 independent measurements; 0.002 n = 7 wells from 2 independent measurements; 0.001 n = 7 wells from 2 independent measurements.
Supplementary Figure 15f	Calibration curve	±30% cutoff	R: 0.2 n = 8 wells from 2 independent measurements; 0.15 n = 8 wells from 2 independent measurements; 0.075 n = 7 wells from 2 independent measurements; 0.025 n = 8 wells from 2 independent measurements; 0.006 n = 8 wells from 2 independent measurements; 0.004 n = 8 wells from 2 independent measurements; 0.002 n = 8 wells from 2 independent measurements; 0.001 n = 8 wells from 2 independent measurements.
Supplementary Figure 15g	Calibration curve	±30% cutoff	E: 0.2 n = 8 wells from 2 independent measurements; 0.15 n = 8 wells from 2 independent measurements; 0.075 n = 8 wells from 2 independent measurements; 0.025 n = 8 wells from 2 independent measurements; 0.006 n = 8 wells from 2 independent measurements; 0.004 n = 8 wells from 2 independent measurements; 0.002 n = 8 wells from 2 independent measurements; 0.001 n = 8 wells from 2 independent measurements.

**Fig. no.**	**Experiment description**	**Statistical Methods**	**Sample numbers/Experimental replicates**
Supplementary Figure 15h	Calibration curve	±30% cutoff	His: 0.2 n = 8 wells from 2 independent measurements; 0.15 n = 8 wells from 2 independent measurements; 0.075 n = 8 wells from 2 independent measurements; 0.025 n = 8 wells from 2 independent measurements; 0.006 n = 8 wells from 2 independent measurements; 0.004 n = 7 wells from 2 independent measurements; 0.002 n = 8 wells from 2 independent measurements; 0.001 n = 7 wells from 2 independent measurements.
Supplementary Figure 15i	Calibration curve	±30% cutoff	buffer: 0.2 n = 8 wells from 2 independent measurements; 0.15 n = 8 wells from 2 independent measurements; 0.075 n = 7 wells from 2 independent measurements; 0.025 n = 8 wells from 2 independent measurements; 0.006 n = 12 wells from 3 independent measurements; 0.004 n = 11 wells from 3 independent measurements; 0.002 n = 11 wells from 3 independent measurements; 0.001 n = 9 wells from 3 independent measurements.
Supplementary Figure 15j	Calibration curve	±30% cutoff	buffer for dilute phase analysis: 0.2 n = 8 wells from 2 independent measurements; 0.15 n = 8 wells from 2 independent measurements; 0.075 n = 8 wells from 2 independent

Fig. no.	Experiment description	Statistical Methods	Sample numbers/Experimental replicates
			measurements; 0.025 n = 8 wells from 2 independent measurements; 0.006 n = 7 wells from 2 independent measurements; 0.004 n = 8 wells from 2 independent measurements; 0.002 n = 5 wells from 2 independent measurements; 0.001 n = 8 wells from 2 independent measurements.
5a	Reaction kinetics analysis	±25% cutoff	**R2H/E:** 0.3 n = 12 wells from 3 independent measurements; 0.5 n = 12 wells from 3 independent measurements; 0.7 n = 28 wells from 8 independent measurements; 0.9 n = 12 wells from 3 independent measurements; 1.1 n = 15 wells from 4 independent measurements<br>**R1H/E:** 0.3 n = 11 wells from 3 independent measurements; 0.5 n = 17 wells from 5 independent measurements; 0.7 n = 22 wells from 6 independent measurements; 0.9 n = 15 wells from 4 independent measurements; 1.1 n = 14 wells from 4 independent measurements<br>**R/E:** 0.3 n = 12 wells from 3 independent measurements; 0.5 n = 11 wells from 3 independent measurements; 0.7 n = 24 wells from 7 independent measurements; 0.9 n = 15 wells from 4 independent

Fig. no.	Experiment description	Statistical Methods	Sample numbers/Experimental replicates
			measurements; 1.1 n = 13 wells from 4 independent measurements.
5c	Encapsulation efficiency	A Two-sample Welch's t-test (two-sided) (OriginPro 2025) was used to compare the two groups. Statistical significance was assessed at α = 0.05. t(54.27)=38.47, with a 95% confidence interval of [19.81, 21.97]. p value is indicated in the figure.	54 condensates and 54 background points from 2 independent measurements.
5d	DLS		n = 9 from 3 independent measurements.
5f	SNARF-1 ratiometric pH probe	A Two-sample Welch's t-test (two-sided) (OriginPro 2025) was used to compare the two groups. Statistical significance was assessed at α = 0.05. $t_{condensates}(32.78)= -15.42$, with a 95% confidence interval of [-0.20, -0.13]. $t_{dilute}(36.57)= 6.15$, with a 95%	Detailed in Table S6. For calibration in Zn^{2+}-free buffer: n = 5 positions in the well for each pH value.

Fig. no.	Experiment description	Statistical Methods	Sample numbers/Experimental replicates
		confidence interval of [0.10, 0.26].<br>p values are indicated in the figure.	
5h-i	FRAP,<br>% recovery and $t_{1/2}$	A Two-sample Welch's t-test (two-sided) (OriginPro 2025) was used to compare the two groups.<br>Statistical significance was assessed at $\alpha = 0.05$.<br>$t(13.10) = -12.28$, with a 95% confidence interval of [-5.97, -4.25].<br>p value is indicated in the figure.	**R2H/E:** 13 condensates from 2 independent measurements.<br>**R/E:** 13 condensates from 2 independent measurements.
Supplementary Figure 16	Reaction kinetics analysis for 60 min		Condensates: Representative results of n = 4 wells. This was repeated three times.<br>Buffer: n = 3 wells.
Supplementary Tables 2-4	Turbidity measurements	NA	Representative results of n = 4 wells. This was repeated twice.
Supplementary Table 5	V_0 at 0.7 mM 4-MU-Ac	±25% cutoff<br>For buffer samples- no cutoff was applied	**R2H+0.67 mM $ZnCl_2$:** n = 11 wells from 3 independent measurements.<br>**E2H+0.67 mM $ZnCl_2$:** n = 11 wells from 3 independent measurements.<br>**Buffer+0.20 mM $ZnCl_2$:** n = 16 wells from 4 independent measurements.

Fig. no.	Experiment description	Statistical Methods	Sample numbers/Experimental replicates
			**Buffer+0.33 mM $ZnCl_2$:** n = 12 wells from 3 independent measurements. **Buffer+0.67 mM $ZnCl_2$:** n = 12 wells from 3 independent measurements.
Supplementary Table 6	SNARF-1 pH values determination		R/E: 3 independent measurements. Detailed in the table
Supplementary Table 7	Kinetic parameters	±25% cutoff	R2H: 0.3 n = 12 wells from 3 independent measurements; 0.5 n = 12 wells from 3 independent measurements; 0.7 n = 16 wells from 4 independent measurements; 0.9 n = 12 wells from 3 independent measurements; 1.1 n = 12 wells from 3 independent measurements.
Supplementary Table 8	V_0 at 0.7 mM 4-MU-Ac	±25% cutoff For buffer samples- no cutoff was applied	**R1H:** n = 12 wells from 3 independent measurements. **R:** n = 12 wells from 3 independent measurements. **E:** n = 12 wells from 3 independent measurements. **His:** n = 12 wells from 3 independent measurements. **Buffer:** n = 16 wells from 4 independent measurements. **R2H/E dilute phase:** n = 16 wells from 4 independent measurements. **R1H/E dilute phase:** n = 16 wells from 4 independent measurements.

Fig. no.	Experiment description	Statistical Methods	Sample numbers/Experimental replicates
			**R/E dilute phase:** n = 15 wells from 4 independent measurements.

Supplementary Table 11. Experimental replicates and statistical methods applied on data.
