# Supplementary material for: Metal-dependent and metal-free mechanisms of peptide condensate catalysts
Source: Nat Commun. 2026 Mar 28;17:4548. doi: 10.1038/s41467-026-71117-4 (PMC13194831; doi:10.1038/s41467-026-71117-4)
Supplement: Supplementary file 3 — Description of Additional Supplementary Files [file 41467_2026_71117_MOESM3_ESM.pdf]

Description of Additional Supplementary Files

File Name: Supplementary Video 1
Description: CLSM time-lapse imaging of coalescence of 0.5 mM R2H/E2H with 0.20 mM $ZnCl_2$. Scale bar, 5 µm. Shown from a single independent experiment.

File Name: Supplementary Video 2
Description: CLSM time-lapse imaging of coalescence of 0.5 mM R2H/E2H with 0.33 mM $ZnCl_2$. Scale bar, 5 µm. Shown from a single independent experiment.

File Name: Supplementary Video 3
Description: CLSM time-lapse imaging of coalescence of 0.5 mM R2H/E2H with 0.67 mM $ZnCl_2$. Scale bar, 5 µm. Shown from a single independent experiment.

File Name: Supplementary Video 4
Description: CLSM time-lapse imaging of coalescence of 3 mM R2H/E. Scale bar, 5 µm. Shown from a single independent experiment.

File Name: Supplementary Video 5
Description: CLSM time-lapse imaging of coalescence of 3 mM R/E. Scale bar, 5 µm. Shown from a single independent experiment.
